# Coffee biowaste valorization within circular economy: an evaluation method of spent coffee grounds potentials for mortar production

**DOI:** 10.1007/s11367-021-01968-0

**Published:** 2021-09-19

**Authors:** Giada La Scalia, Manfredi Saeli, Pier Paolo Miglietta, Rosa Micale

**Affiliations:** 1 Department of Engineering, University of Palermo, Viale delle Scienze, Bld 8, Palermo, Italy; 2 Department of Architecture, University of Palermo, Viale delle Scienze, Bld 8-14, Palermo, Italy; 3 Department of Biological and Environmental Sciences and Technologies, University of Salento, Via per Monteroni, Lecce, Italy

**Keywords:** Coffee supply chain, Life cycle approach, Food waste valorization, Circular economy, Construction material, Multi-criteria analysis, Sustainability

## Abstract

**Purpose:**

Spent coffee grounds (SCG) are biowastes extensively generated within the coffee supply chain. Nowadays, their disposal represents an increasing environmental concern due to its toxicity and organic nature. With the estimated increase of coffee production and consumption in the upcoming years, there is an imperative need to find a proper reverse option, along with a novel industrial application, which allows for the valorization of this coffee by-product within a circular economy perspective. This study aims at investigating a potential reuse of spent coffee grounds to produce novel construction materials to be used for sustainable buildings.

**Methods:**

After having illustrated the forward flows within the coffee life cycle and the potential reverse flow options, an evaluation method based on multi-criteria analyses was elaborated to test not only the technical but also the environmental and economic performances of novel materials originating from the incorporation of SCG as an aggregate in natural hydraulic lime and geopolymer-based mortars. Moreover, we focus on the reuse of another waste streams— biomass fly ash—deriving from the paper-pulp industry, rarely investigated in both traditional construction applications and in geopolymer manufacture. The two (geopolymer- and lime-based) mortar typologies are here studied and compared as potential green material for applications in construction, with satisfying engineering performance and high insulation attitude, giving a new life to a common organic waste. Consequently, we compare eight formulations by means of multi-criteria approaches that are nowadays claimed as a useful and effective decision aiding support instrument to assess the development of new sustainable construction materials. They permit to consider simultaneously some controversial and often uncertain aspects like technological (as the usual scientific studies do), environmental, and economic (more difficult to easily approach and evaluate). For this purpose, in this paper, we have analyzed the performance of the novel bio-composite mortars using VIKOR and TOPSIS methods to rank a set of alternatives according to various evaluation criteria that often conflict one with each other.

**Results:**

Results show that adding spent coffee grounds can efficiently improve the technical and sustainable performances of the novel mortars for different applications in the building sector. The presence of SCG increases water absorption and improves the insulation performance along with an environmental impact reduction. The considered technological properties are highly promising—such as the improvement in thermal insulation. In particular, even the addition of only 5% SCG leads to a significant reduction of the thermal conductivity and consequently to a greater insulating performance.

**Conclusions:**

To date, most of the available literature on recycling SCG in construction materials do not consider mortar-based applications and, moreover, nor multi-criteria approaches. Therefore, our study proposes itself as an innovative track solution to food waste management lowering the employment of non-renewable natural resources and the costs associated to construction material production. At the same time, a novel and innovative way of such waste disposal is suggested, pursuing the sustainability and substantially reducing the environmental impact of construction and building materials. This study is a fundamental step in assessing the applicability of our designed and produced materials and its potentials to be produced at an industrial scale.

## Introduction

Coffee is one of the most consumed and popular beverages drunk worldwide. Despite social distancing, required by the COVID-19 pandemic spreading, is limiting de facto the common out-of-home coffee consumption and the global economy recover, the consumption of coffee-based beverages during 2021 is estimated to increase by 1.3%, amounting to about 166 million bags (9978 million kg) (ICO 2021). Nevertheless, the coffee industry is globally responsible for producing a great quantity of waste, mainly “coffee silver skin” and spent coffee grounds (SCGs) (Mussatto et al. 2011; Murthy 2012). The latter are mainly generated from the brewing process or by the soluble coffee industry. It is reported that approximately 6 million tons of SCGs are produced every year worldwide (Getachew and Chun 2017). The traditional disposal procedure of this residue is in landfill; however its dispersion into the environment should be strongly prevented due to its potential toxicity and organic nature (Ktori et al. 2018; Massaro Sousa and Ferreira 2019). Indeed, disposing SCGs in landfills is unsafe, as for most organic wastes, because the risk of spontaneous combustion is quite high, and, moreover, an excessive production of harmful methane and carbon dioxide may occur (Massaro Sousa and Ferreira 2019) contributing to the overall atmospheric pollution.

In this context, various industries decided to change their development model, focusing on the use of sustainable as well as renewable, eco-friendly, and cheap resources, like SCGs, adopting a circular economy approach (Karmee 2018; Son et al. 2018; Nguyen et al. 2019; Dattatraya Saratale et al. 2020).

Worldwide, there has been growing attention and interest among researchers and industries to utilize re-usable resources aimed at generating more sustainable materials to be applied in construction. Many studies are focused on developing sustainable construction and building materials, especially as an alternative to the Portland cement claimed to be one of the most polluting industries in the sector (Saberian et al. 2021).

Agri-food by-products and biowaste reuse shows great potentialities in the construction industry. As shown by much of the literature, life cycle methodologies underpin Circular Economy strategies but also highlight some weaknesses (Notarnicola et al. 2016; Peña et al. 2021) which can be overcome through the proper use of multi-criteria approaches. Recent studies, in fact, have demonstrated that multi-criteria approaches are a useful and effective decision aiding support tools to assess the potentials of new sustainable construction materials (Moretti et al. 2017; Kurda et al. 2019; Saeli et al. 2020). In the selection of novel materials, the need to consider simultaneously contradictory and often uncertain aspects—like technological, environmental, and economic ones—makes the multi-criteria approach extremely suitable (Micale et al. 2017), even if to date, most of the available literature on recycling SCG in construction materials has not considered in multi-criteria approaches (Saberian et al. 2021).

This study aimed at examining the performance of novel mortars obtained by incorporating various amounts of SCG as aggregate in substitution (volume %) to the traditional sand. For this purpose, two different typologies of binder were considered: a green geopolymer that employs itself an industrial waste as raw material, biomass fly ash (BFA), and a traditional hydraulic lime. The application of multi-criteria approach is proposed to provide further knowledge on the development of an alternative SCG recycle option to ordinary disposal in landfill, the potentials of the sustainable construction materials based on SCG reuse. In order to evaluate the industrial feasibility of the proposed solution from environmental and economic perspectives, the VIKOR (VIekriterijumsko KOmpromisno Rangiranje) (Opricovic 1998) and the TOPSIS (Technique for Order of Preference by Similarity to Ideal Solution) (Hwang and Yoon 1981) multi criteria decision-making methods were implemented to rank a set of alternatives according to various evaluation criteria often conflicting with one another (Opricovic and Tzeng, 2004; Chu et al. 2004; Opricovic and Tzeng 2007; La Fata et al. 2021).

In the light of the circular economy (CE) approach, the coffee biowaste valorization presented in this study is at a preliminary stage. Consequently, future studies should focus on the techno-economic analysis along with the industrial scale productivity and the producible bioproduct feasibility.

The remainder of this paper is organized as follows. Section 2 presents the coffee life cycle and its circular economy potentials. Section 3 outlines the study methodological approach, providing also an overview of the evaluation criteria. Section 4 presents the case study and discusses the findings, before the study is concluded in Sect. 5.

## Coffee life cycle and circular economy

Coffee is nowadays considered one of the most appreciated beverages internationally. It is grown in over 70 countries, and it immediately follows petroleum as the second most globally traded commodity (Crossley et al. 2020).

**Fig. 1:**
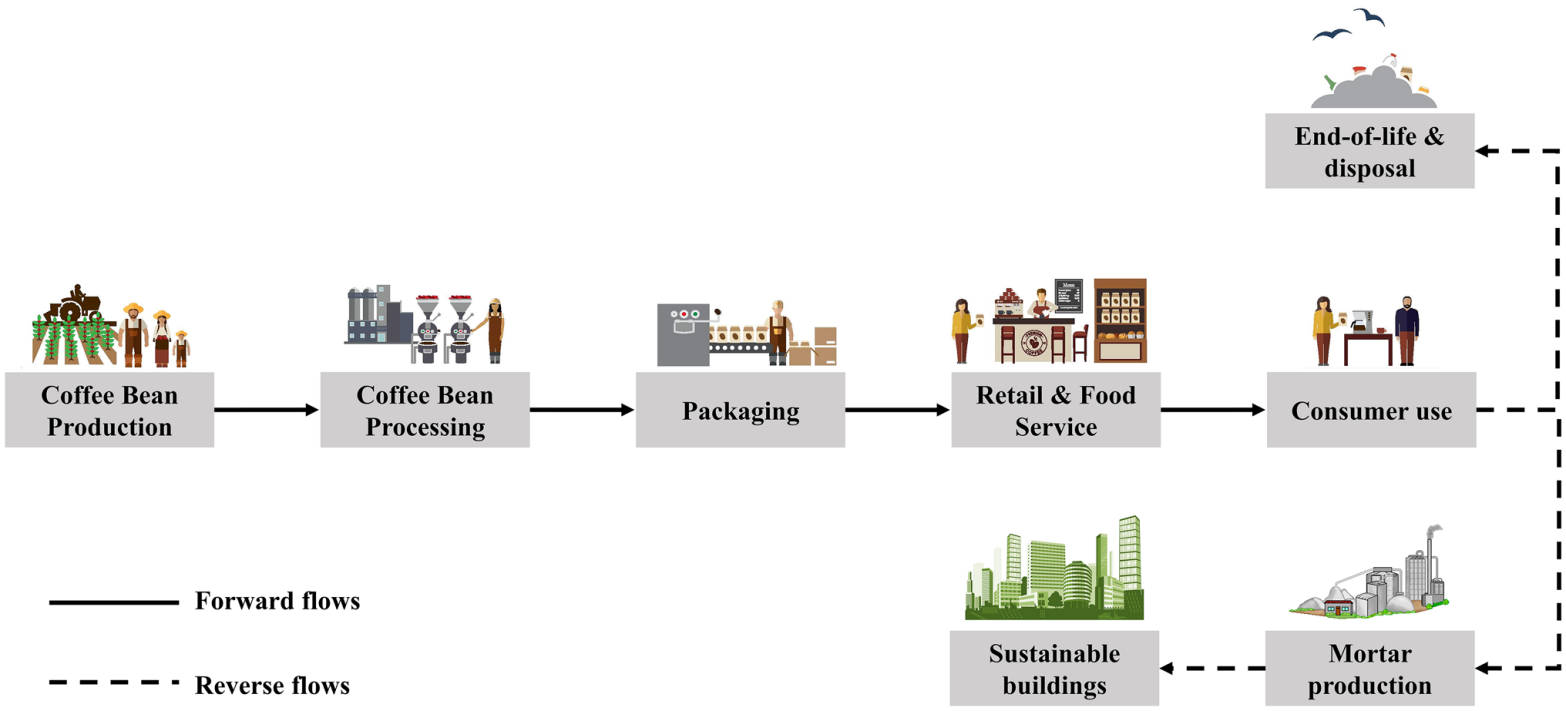
Forward and reverse flows within the coffee life cycle

Cultivated mainly in tropical climates, the coffee life cycle begins with freshly picked coffee cherries (Fig. 1). As described by Murthy and Naidu (2012) and Figueroa et al. (2016), coffee beans are then processed through (i) the dry process which uses sunlight to simply dry the cherry, allowing the “coffee bean” to be removed, and/or (ii) the more complex wet process, based on the use of water and pressure, which allows the coffee pulp to be removed, obtaining clean coffee beans, but also highly polluted wastewater.

After either process is completed, the beans are roasted, which subsequently results in the bean shedding its thick protective coating or “coffee silver skin.” Finally, the beans are packaged and distributed to retail and food services, cafes, and households where the beans are ground and brewed, resulting in the production of the final SCG biowaste (Fig. 1).

Fifty percent of the coffee beverage impacts refers to the life cycle stages under the control of producers and their suppliers (coffee bean production, processing, packaging, and distribution) and the other 50% under the control of users (retail and food services, consumers, and final disposal) (de Figueiredo Tavares and Mourad 2020).

The forward flows within the whole coffee life cycle, from coffee cherry to coffee grounds, have gained more spread over time resulting in more production and subsequently more damage. However, although the entire lifetime of the coffee supply chain produces, each year, a large amount of biowaste (Kovalcik et al. 2018) whose value is widely discussed in the literature (Musatto et al. 2011; Acevedo et al. 2013; Luz et al. 2018; Franca and Oliveira 2019; Schmidt Rivera et al. 2020), SCGs certainly represent the largest percentage.

Since the global demand for coffee-based beverages constantly increases, subsequently SCGs will also continue to grow, proving detrimental to the environment. SCG physical properties and chemical compositions are thus fundamental in exploring their potential to be used within a circular economy context (Rangarajan 2019).

Apart from the more established SCG recycling applications in various industrial sectors, especially that of soil conditioner, compost, and fertilizers and that of bio-based additives (e.g., Ronga et al. 2016; Santos et al. 2017; Najdanovic-Visak et al. 2017; Girotto et al. 2018), attentions have been concentrated on the opportunity to avoid end-of-life and disposal of SCGs, proposing an alternative potential reverse flow (Fig. 1) within the coffee supply chain aimed at using SCGs in the production of construction material for sustainable buildings and civil engineering applications.

In fact, the main purposes of the current study are (i) to investigate the SCG incorporation as an aggregate in construction materials, allowing for the valorization of coffee biowaste within a circular economy approach, and (ii) to develop an evaluation method of SCG potential for mortar production also based on the life cycle approach towards sustainable buildings.

## Material and methods

### Spent coffee grounds

**Fig. 2:**
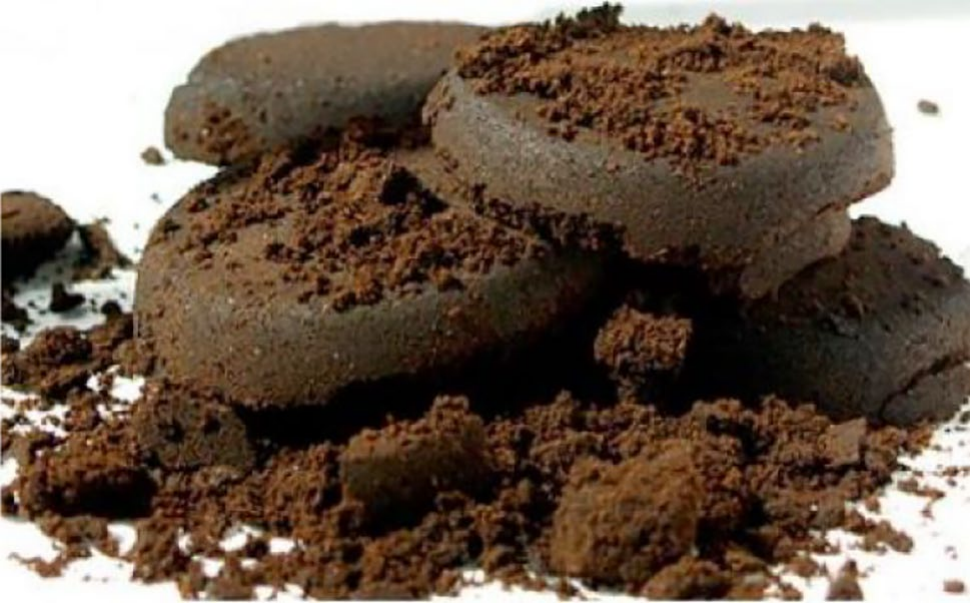
Spent coffee grounds

**Fig. 3:**
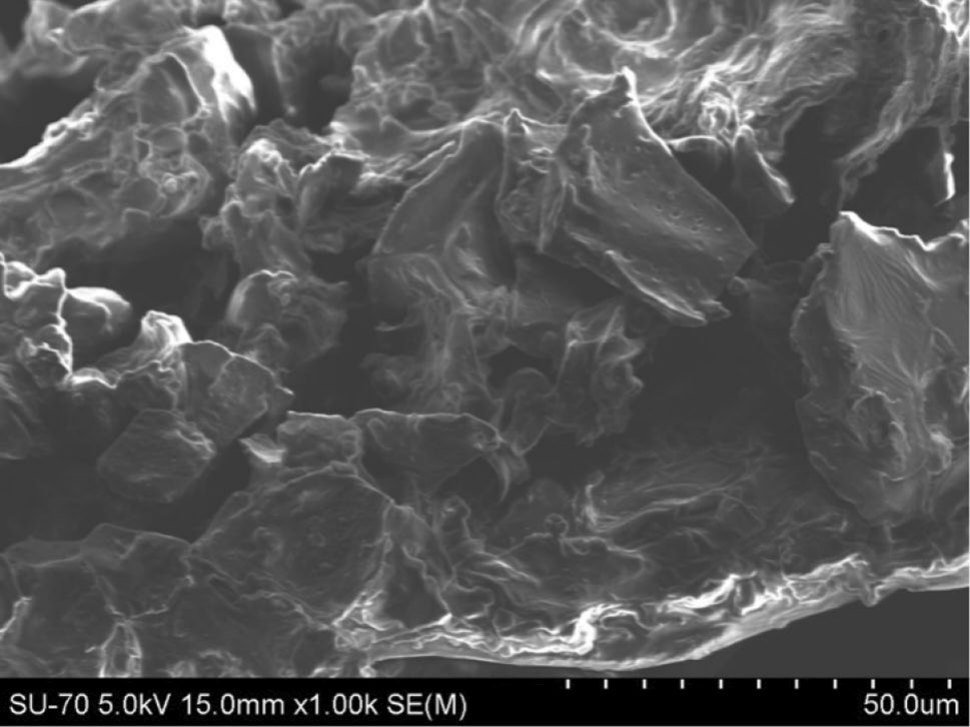
Micrograph image of SCG

The SCGs used in this study were illustrated in Fig. 2 and obtained as a domestic waste deriving from the ordinary *moka* Italian coffee makers, and—prior to use—it was dried naturally until the material had almost no moisture content. The used SCG particle size distribution was measured by laser diffraction (Coulter LS230 analyzer, Fraunhofer method and Polarization Intensity Differential Scattering) and the median particle dimension resulted 243.7 µm. Based on the Brunauer–Emmett–Teller analysis (BET), the SCG surface area resulted to be 0.48 m^2^/g. The bulk density of the dried particle was 400 kg/m^3^, and, compared with the density of lightweight aggregates, SCGs might be exploited in lightweight concrete production with unit weight less than 1900 kg/m^3^ (ACI 213 2003). In Fig. 3 the micro-morphological features (SEM) of SCGs are shown; the image reveals a corrugated surface with jagged and crumpled particles, but a compact matrix.

### Mortar specimens

In this study, mortars employing two different typologies of hydraulic binder—geopolymer (GP) and natural hydraulic lime (NHL)—and manufactured with various quantities of SCG were considered and compared.

The first mortar typology was prepared from an innovative GP binder designed and manufactured according to previous studies of authors (Saeli et al. 2017, 2019a, 2019b). GP is a hydraulic alkali-activated binder usually investigated as a possible greener substitute to Ordinary Portland Cement. In this study, the formulation used foresees a mixture of BFA and metakaolin (MK) as a solid source of alumina and silica, in a ratio BFA/MK equal to 70:30 wt.%. The BFA used is a solid waste deriving from the paper-pulp industrial Kraft process. It is important to highlight that in this study the BFA largely substituted the MK as a source of alumina and silicate, improving the material’s sustainability. It is obtained by reusing a waste product, saving natural resources (kaolin used to prepare the MK), eliminating the carbon footprint associated with MK manufacturing (extraction, transport, calcination, etc.), minimizing the financial commitment for waste disposal, etc. The alkaline activator was prepared mixing sodium silicate and sodium hydroxide as in Saeli (2019a). Details of the raw materials used and GP binder characterization are presented in the mentioned references. The second mortar typology was prepared from NHL as a binder, produced by Axton and furnished in powder. In this study, NHL is used as reference being a traditional commonly used material in construction.

The used aggregate consisted in a mixture of a natural siliceous sand (particle dimension ranging 0–4 mm) and SCG. The aggregate mix design is shown in Table 2. For both the considered mortars (NHL-based and GP-based), the selected binder/aggregate ratio was constant and equal to 1:3 in volume. Virgin NHL-mortar (NHL_0, without SCG) was designed in order to present the same workability (spread) of the reference GP-mortar (GP_0, without SCG) (cfr. Table 1). That resulted for a water/NHL ratio equal to 1:1. Consequently, the resulting mortars’ water/solid ratios resulted 0.155 and 0.196 for NHL-mortar and GP-mortar, respectively.

**Table 1:** Main characteristics of the used reference mortars

Property	Measured value	
	NHL-mortar	GP-mortar
Workability (spread by flow table) [cm]	21	21
Water/solid ratio	0.155	0.196
Bulk density [Kg/m^3^]	1706	1832
Water absorption [%]	11	13
Compressive strength [MPa]	14.10 ± 0.18	21.66 ± 1,91
Flexural strength [MPa]	2.76 ± 0.016	4.08 ± 0.72

The relevant features of the reference NHL-mortar and GP-mortar are summarized in Table 1.

In this study, the main properties and performance of the NHL-mortars and GP mortars manufactured implementing the SCG waste quantities were analyzed, compared, and ranked by using multi-criteria decision-making methodologies. The foreseen use is a plastering application for novel architectural finishing with insulating performance. Mortar mix design was formulated in order to improve the material performance, along with its sustainability, by adding increasing quantities of SCG to substitute the sand. More particularly, the aggregate was prepared by substituting increasing quantities of sand with SCG (5%, 10%, 17.5% in volume) until the material became too viscous to be appropriately mixed (cfr. flow table test). Eight different formulations were considered as listed in Table 2. Here, the aggregate mix design is presented in volumetric parts extrapolated from the whole mortars mix designs (binder—GP or NHL—and liquid, water or alkaline activator, parts are not shown as the two products derive from different manufacturing processes). Also, the relative volume percentages among sand and SCG are shown.

**Table 2:** Mortar formulations: aggregate mix design

N	ID	Substitution [volume %]	Mix design—aggregate			
			Volume [%]		Volumetric part	
			Sand	Coffee	Sand	Coffee
1	NHL_0	0	100	0	3	0
2	NHL_5	5	95	5	2.85	0.15
3	NHL_10	10	90	10	2.7	0.3
4	NHL_17.5	17.5	82.5	17.5	2.475	0.525
5	GP_0	0	100	0	3	0
6	GP_5	5	95	5	2.85	0.15
7	GP_10	10	90	10	2.7	0.3
8	GP_17.5	17.5	82.5	17.5	2.475	0.525

The testing specimens were produced in accordance with EN 998–2: 2016 and UNI EN 196:1: 2016. In the case of the GP-mortar, the procedure was derived from the common method for traditional cementitious materials. The manufacture process is simple and reproducible and was performed at environmental conditions (25 °C, 65% RH), avoiding external fonts of heat, as frequently done in GP-manufacturing, making the material more sustainable. The first step of the manufacturing process foresees the precursors’ preparation: the solid alumino-silicate source (BFA + MK) and the liquid alkaline activator. Precursors are mixed for 9 min to produce the GP binder. Then, the aggregate (sand, eventually admixed with the SCG waste) is added to the slurry and mixed for 1 min to ensure uniformity. At this stage the mortar is produced. The slurry was then poured in standard metallic molds (UNI EN 1015–11:^2007^), vibrated for 2 min, sealed until hardening (1 day). Samples were then cured until testing (28th day), in accordance with the standardized testing procedure. NHL-mortar was produced mixing the lime with water for 30 s. Here, the used water was taken from the municipal aqueduct in accordance with UNI EN 1008:^2003^ and UNI EN 206–1: 2006. Subsequently the aggregate (sand or sand + SCG) was added and mixed for other 210 s (total mixing time was 240 s). The slurry was then poured into the same standard metallic molds, vibrated for 2 min, sealed for 7 days (2 days in the mold and 5 days demolded), in accordance with the relevant standard; specimens were then cured for other 28 days until testing (Boresi et al. 1993).

### Evaluation criteria

With the aim of selecting the best SCG quantity to substitute the sand, different evaluation criteria have to be taken into account depending on the particular application for which they are intended (Saeli et al. 2020; Chudley 2016). This choice is related to the freshness and hardness properties of the materials (e.g., workability, bulk density, compressive strength, etc.) and the environmental and economic impacts.

The evaluation criteria selected in this study were the following:

- Uniaxial compressive strength [MPa] (UCS) indicates the material resistance to compressive loads. According to EN 998–2:^2016^, it was measured using a universal testing machine (Shimadzu, AG-25TA), equipped with a 250 kN load cell running at 0.5 mm/min displacement rate. The given value is the mean from three tests.
- Axial strain [%] (AS) is the specimen strain at rupture by compression (cf. UCS) and was calculated as the quotient between the displacement at rupture and the initial specimen length.
- Flexural strength [MPa] (FS) indicates the maximum resistance by pure flexion. It was determined using a universal testing machine (Shimadzu, AG-25TA), equipped with a 20 kN load cell running at 0.5 mm/min displacement rate. The given value is the mean calculated from three tests.
- Bending deflection [mm] (BeD) represents the degree to which the simply supported specimen (10 cm distance) is displaced under the load applied in the center.
- Bulk density [kg/m^3^] (BuD) represents the geometric mean value calculated from three different specimens cured for 28 days.
- Water absorption by immersion [%] (WAI) indicates the quantity of water—by weight variation (ΔP/P %)—absorbed by a specimen in consequence of full immersion in water. The value is the average from three tests.
- Workability [cm] (W) indicates the consistency of the just produced slurry returning the product attitude to be mixed until reaching homogeneity and consequently used conveniently. It is strictly related to the slurry fluidity (or conversely viscosity) and the specific considered application. It was evaluated by flow table test in accordance with EN 1015–3:^1999^ and expressed in terms of spread.
- Thermal conductivity [W/m·K] (TC) indicates the heat transfer rate through the specimens’ surfaces and was measured using a calorimeter HFM-CT 1000, in accordance to ISO 6946: (2017).
- Sustainability [qualitative] (S) intends the environmental and economic impact reduction in terms of waste disposal. As SCG contains many harmful compounds—such as polyphenols, tannins, and caffeine that may pollute the environment—the greater the amount of SCG used in the material, the lower the costs for its disposal and the greater raw materials saving (less the sand usage). This criterion is qualitative, and it is expressed in the range [1; 10].

### The VIKOR method

The traditional VIKOR method, introduced by Opricovic (1998), is a multi-criteria decision-making approach useful for dealing with complex systems (Chatterjee and Chakraborty 2016; Opricovic and Tzeng 2004). This method allows for a ranking of a set of options when the evaluation criteria are in conflict with each other and suggests a compromise solution (Opricovic and Tzeng 2007). VIKOR is built on an aggregating function that expresses the closeness to the ideal solution, and it considers the relative criteria importance and a balance between individual and total satisfaction (San Cristobal 2011). The approach needs the following input data: (i) a decision matrix *F* where the element *f*_*ij*_ represents the rating of the alternative *i* (*i* = 1, …, *n*) with respect to criterion *j* (*j* = 1, …, *m*) and (ii) the weights of criteria *w*_*j*_.

The approach follows the subsequent steps:

- Best and worst scores (i.e. ${f}_{j}^{*}$ and ${f}_{j}^{-}$) will be determined for every criterion *j*. In detail, for benefits criteria
  1 $${f}_{j}^{*}={max}_{i}{f}_{ij}\quad j=1, \dots , m$$
  2 $${f}_{j}^{-}={min}_{i}{f}_{ij}\quad j=1, \dots , m$$

whereas for costs criteria:

3 $${f}_{j}^{*}={min}_{i}{f}_{ij}\quad j=1, \dots , m$$

4 $${f}_{j}^{-}={max}_{i}{f}_{ij}\quad j=1, \dots , m$$

- Calculation of *S*_*i*_ and *Q*_*i*_ for every alternative *i*:
  5 $$S_i={\textstyle\sum_{j=1}^m}\left[\frac{w_j\cdot\left(f_j^\ast-f_{ij}\right)}{\left(f_j^\ast-f_j^-\right)}\right]i=1,\dots,n$$
- 6 $$Q_i=max_j\left[\frac{w_j\cdot(f_j^\ast-f_{ij})}{f_j^\ast-f_j^-}\right]i=\;1,\dots,n$$
  Calculation of the *R*_*i*_ value for every alternative *i*:
  7 $$R_i=\nu\cdot\frac{\left(S_i-S^\ast\right)}{\left(S^--S^\ast\right)}+(1-\nu)\cdot\frac{(Q_i-Q^\ast)}{(Q^--Q^\ast)}\quad i=1,\dots,n$$

where

8 $${S}^{*}={min}_{i}{S}_{i}$$

9 $${S}^{-}={max}_{i}{S}_{i}$$

10 $${Q}^{*}={min}_{i}{Q}_{i}$$

11 $${Q}^{-}={max}_{i}{Q}_{i}$$

and 0 $\le \nu \le 1$. In particular, when $\nu$ is small (i.e., $\nu <0.5$), the individual regret is emphasized, whereas as $\nu$ increases (i.e., $\nu >0.5$), the strategy of maximum group utility is favored. The value $\nu =0.5$ represents, instead, the consensus. This value impacts the alternatives’ final ranking, it is set by experts, and generally the value equal to 0.5 is considered (Chatterjee and Chakraborty 2016).

- Sorting of the alternatives according to *S*_*i*_, *Q*_*i*_ and *R*_*i*_ is from the lowest to the highest value. The alternative a^1^ (i.e., the first alternative in the *R*_*i*_ list of the ranking) is a compromise solution if both the following conditions are fulfilled:

1. Acceptable advantage: where is the alternative ranked second in the Ri ranking list.
2. Acceptable stability in decision-making: alternative a1 is also the best solution in the Si or/and Qi ranking list.

If just one of the mentioned conditions (1) and (2) is not satisfied, then a set of compromise solutions is given. The set of compromise solutions is determined as follows:

- *a*^*1*^ and *a*^*2*^ if only the condition (2) is not satisfied
- *a*^*1*^*, a*^*2*^*, …, a*^*z*^ if the condition (1) is not satisfied where *a*^*z*^ is the last alternative placed in the *R*_*i*_ ranking list for which.

## Results and discussions

In this paper the alternatives are the samples reported in Table 2, whereas the evaluation criteria are depicted in Sect. 3.3. The relative importance of the selected criteria was weighed by a board of experts by employing the Delphi technique (Delbecq 1975). The panel of experts was iteratively interviewed asking them to express a judgment of the criteria of relative importance considering the specific application context until an agreement is reached. Considering that the produced samples are intended for architectural finishing with insulating performance, in Table 3 for every evaluation criterion, the preference versus (i.e., if the criterion is to be maximized or minimized) and the weights are reported:

**Table 3:** Criteria weights and their preference versus

Criteria	**UCS**	**AS**	**FS**	**BeD**	**BuD**	**WAI**	**W**	**TC**	**S**
	max	min	max	min	min	min	max	min	max
Weights	0.117	0.095	0.119	0.099	0.100	0.098	0.105	0.111	0.156

The decision matrix is reported in Table 4. The evaluation of workability criterion is represented through the following trapezoidal membership function:

**Table 4:** Decision matrix of the proposed case study

	**UCS** [MPa]	**AS** [%]	**FS** [MPa]	**BeD** [mm]	**BuD** [kg/m^3^]	**WAI** [%]	**W** [cm]	**TC** [W/m·K]	**S** [qualitative]
GP_0	21.660	2.65	4.08	0.460	1832	13.00	23.0	575.000	8.00
GP_5	18.668	2.88	3.64	0.430	1794	13.43	20.3	568.000	8.50
GP_10	20.826	1.92	3.23	0.340	1739	13.83	15.0	521.000	9.00
GP_17.5	10.633	0.59	2.58	0.270	1648	16.49	10.0	470.000	9.75
NHL_0	14.100	2.73	2.76	0.400	1706	11.11	21.0	551.388	7.00
NHL_5	12.400	2.81	2.78	0.230	1474	11.54	20.0	460.518	7.50
NHL_10	2.850	2.82	1.53	0.082	1468	18.18	18.5	292.104	8.00
NHL_17.5	2.210	3.12	0.94	0.290	1581	19.49	10.0	364.031	8.75

12 $$f_{ij}=\left\{\begin{array}{cc}\frac{(x-10)}8&10\leq x<18\\1&18\leq x\leq22\\\frac{(30-x)}8&22<x\leq30\end{array}\right.$$

where x is the measurement of the workability for every sample obtained by means of the flow table test. With regard to the sustainability criterion, the panel of experts was queried asking them to express a judgment belonging into the interval [1; 10] in which 10 is the best value.

Considering the weights and the decision matrix reported in Tables 3 and 4, respectively, the VIKOR method was implemented. The method was applied considering different values of the parameter $\nu$ starting from 0 to 1. In particular, $\nu = 1$ emphasizes the strategy of maximum group utility (i.e., majority rule), whereas $\nu = 0$ is the strategy of minimum individual regret. In Table 5 the values of *R*_*i*_ as a function of parameter $\nu$ are reported:

**Table 5:** Values of *R*_*i*_ as a function of $\nu$

**Alternatives**	${\varvec{\nu}}=\boldsymbol{ }0.0$	${\varvec{\nu}}=\boldsymbol{ }0.1$	${\varvec{\nu}}=\boldsymbol{ }0.3$	${\varvec{\nu}}=\boldsymbol{ }0.5$	${\varvec{\nu}}=\boldsymbol{ }0.7$	${\varvec{\nu}}=\boldsymbol{ }0.9$	${\varvec{\nu}}=\boldsymbol{ }1.0$
GP_0	0.32012	0.32218	0.32629	0.33041	0.33452	0.33864	0.34069
GP_5	0.27862	0.27970	0.28187	0.28404	0.28620	0.28837	0.28945
GP_10	0.00000	0.00190	0.00569	0.00948	0.01327	0.01706	0.01896
GP_17.5	0.22947	0.21811	0.19539	0.17268	0.14996	0.12724	0.11588
NHL_0	1.00000	0.95588	0.86764	0.77940	0.69116	0.60292	0.55880
NHL_5	0.57147	0.51432	0.40003	0.28573	0.17144	0.05715	0.00000
NHL_10	0.35260	0.33467	0.29880	0.26294	0.22707	0.19120	0.17327
NHL_17.5	0.44099	0.49689	0.60869	0.72049	0.83230	0.94410	1.00000

In correspondence with the values of the parameter $\nu$ equal to 0.7, 0.9, and 1, the condition (1) reported in Sect. 4 is not satisfied, and therefore a set of compromise solutions will be proposed. The different rankings obtained are reported in Table 6:

**Table 6:** Ranking with the VIKOR method as function of $\nu$

*#*	${\varvec{\nu}}=0.0$	#	${\varvec{\nu}}=0.1$	#	${\varvec{\nu}}=0.3$	#	${\varvec{\nu}}=0.5$	#	${\varvec{\nu}}=0.7$	#	${\varvec{\nu}}=0.9$	#	${\varvec{\nu}}=1.0$
*1*	GP_10	1	GP_10	1	GP_10	1	GP_10	1	GP_10 GP_17.5	1	GP_10 NHL_5 GP_17.5	1	NHL_5 GP_10 GP_17.5
*2*	GP_17.5	2	GP_17.5	2	GP_17.5	2	GP 17.5						
*3*	GP_5	3	GP_5	3	GP_5	3	NHL_10	2	NHL_5				
*4*	GP_0	4	GP_0	4	NHL_10	4	GP_5	3	NHL_10	2	NHL_10	2	NHL_10
*5*	NHL_10	5	NHL_10	5	GP_0	5	NHL_5	4	GP_5	3	GP_5	3	GP_5
*6*	NHL_17.5	6	NHL_17.5	6	NHL_5	6	GP_0	5	GP_0	4	GP_0	4	GP_0
*7*	NHL_5	7	NHL_5	7	NHL_17.5	7	NHL_17.5	6	NHL 0	5	NHL_0	5	NHL 0
*8*	NHL_0	8	NHL_0	8	NHL_0	8	NHL 0	7	NHL_17.5	6	NHL_17.5	6	NHL_17.5

A ranking of the samples (Table 7) was also performed by applying the TOPSIS method. As this method is not the core of the paper, the formulas can be found in Sciortino et al. (2019).

**Table 7:** C* values and ranking with the TOPSIS method

C*	Ranking
0.6270	GP_10
0.6025	GP_0
0.5894	GP_5
0.5785	NHL_5
0.5283	NHL_0
0.4742	NHL_10
0.4580	GP_17.5
0.2237	NHL_17.5

It is observed that the sample GP_10 resulted to be in the first position with both the considered multi-criteria methods. Nevertheless, as shown in Table 6, as $\nu$ increases a set of compromise solutions exists with the VIKOR (i.e., GP_10 and GP_17.5 for $\nu$ = 0.7 and NHL_5; GP_10 and GP_17.5 for $\nu =0.9$ and $\nu =1$). The sample NHL_0 is in the last position with the VIKOR for $\nu \le 0.5$, instead for $\nu >0.5$, and using the TOPSIS approach, the sample NHL_17.5 is placed in last position.

GP_10 indeed shows a good compromise among the various mix designs between engineering performance and SCG implementation. In fact, it is characterized by a very high mechanical resistance in compression (mortar resistance class M20) and an acceptable bending resistance. It is indisputable that all the GP mortars show a higher resistance than NHLs’, even though all the produced specimens might be used for applications where the mechanical performance plays a greater role, such as in the structural ones. In terms of deformation, GP_10 shows an average deflection and an acceptable strain. Water absorption is not influenced much by the high SCG addition, and the spread on the flow table lets the slurry workable for a suitable mix for finishing application: a suitable workability to be placed onto vertical surfaces. Finally, it shows an implemented thermal insulation making it suitable to improve building energy performance. The great quantity of SCG makes it highly sustainable and innovative in comparison to the virgins and the 5% mixes, and with better overall performances than 15%—mainly showing the great limit of being unworkable, and therefore rejected as the main choice.

## Conclusions

In this study a method for evaluating spent coffee grounds potential as an additive within mortar production was investigated to verify if the coffee life cycle can provide an alternative circular process for this coffee biowaste recycling. More particularly, mortars were produced starting from two different binder typologies: a green geopolymer and an ordinary hydraulic lime. SCGs substituted sand in various proportions within the mortar manufacturing. The aggregate mixtures, whose proportions were accurately designed, gave proper features to the analyzed mortars to be qualified for industrial processing. It was observed that some bio-composites showed improved mechanical characteristics. The presence of SCG increases water absorption and improves the insulation performance along with an environmental impact reduction. The considered technological properties are highly promising—such as the improvement in thermal insulating. In particular, even the addition of only 5% SCG leads to a significant reduction of the thermal conductivity and consequently to a greater insulating performance. Based on the obtained results, it can be stated that spent coffee grounds can be efficiently recycled and employed to produce novel sustainable mortars with suitable mechanical properties and low thermal conductivity which could improve the energy efficiency of buildings. Moreover, SCG could be valorized as a secondary raw material avoiding landfill disposal of non-renewable resources. The proposed circular economy solution represents an innovative life cycle-based food waste management which could reduce the costs of mortar manufacturing and the non-renewable natural resource utilization. Complexity, data availability, lack of priority, and cost were identified as major obstacles for mainstreaming life cycle thinking in the economy. Simplifying tools and approaches to model complex systems, as those adopted for the aim of the present study, have the potential to overcome obstacles for mainstreaming life cycle thinking in the economy (Stucki et al. 2021). Among the life cycle-based approaches, most helpful to support decision-makers, multi-criteria decision analysis is particularly appreciated to enhance agri-food waste in the building material field.

Future research developments should concern the analysis of other sets of bio-based materials to evaluate their possible usage in the construction sector, also including further evaluation criteria. In order to make the presented approach a valid decision-making support tool useful for real implementations of CE strategies, a sensitivity analysis could be carried out to check uncertainty levels.

## Data Availability

The authors declare that the data supporting the findings of this study are available within the article.
